# Partial Least-Squares Regression as a Tool to Retrieve Gas Concentrations in Mixtures Detected Using Quartz-Enhanced Photoacoustic Spectroscopy

**DOI:** 10.1021/acs.analchem.0c00075

**Published:** 2020-07-17

**Authors:** Andrea Zifarelli, Marilena Giglio, Giansergio Menduni, Angelo Sampaolo, Pietro Patimisco, Vittorio M. N. Passaro, Hongpeng Wu, Lei Dong, Vincenzo Spagnolo

**Affiliations:** † State Key Laboratory of Quantum Optics and Quantum Optics Devices, Institute of Laser Spectroscopy, Shanxi University, Taiyuan 030006, P. R. China; ‡ PolySense Lab—Dipartimento Interateneo di Fisica, University and Politecnico of Bari, CNR-IFN, Via Amendola 173, 70125 Bari, Italy; § Collaborative Innovation Center of Extreme Optics, Shanxi University, Taiyuan 030006, P. R. China; ∥ Photonics Research Group, Dipartimento di Ingegneria Elettrica e dell’Informazione, Politecnico di Bari, Via Orabona 4, Bari 70126, Italy

## Abstract

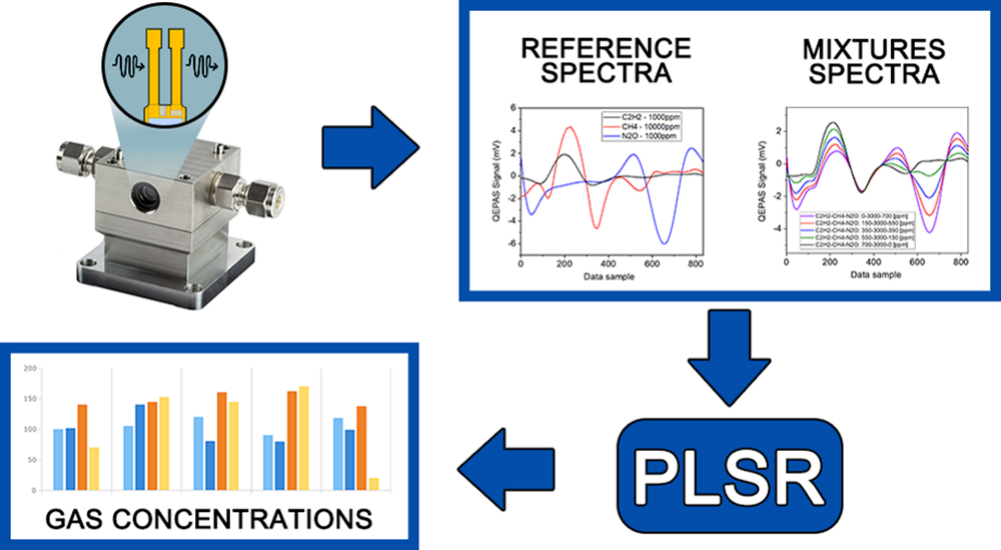

We report on a statistical tool based on partial least-squares regression (PLSR) able to retrieve single-component concentrations in a multiple-gas mixture characterized by spectrally overlapping absorption features. Absorption spectra of mixtures of CO–N_2_O and mixtures of C_2_H_2_–CH_4_–N_2_O, both diluted in N_2_, were detected in the mid-IR range by exploiting quartz-enhanced photoacoustic spectroscopy (QEPAS) and using two quantum cascade lasers as light sources. Single-gas reference spectra of each target molecule were acquired and used as PLSR-based algorithm training data set. The concentration range explored in the analysis varies from a few parts-per-million (ppm) to thousands of ppm. Within this concentration range, the influence of the gas matrix on nonradiative relaxation processes can be neglected. Exploiting the ability of PLSR to deal with correlated data, these spectra were used to generate new simulated spectra, i.e., linear combinations of the reference ones. A Gaussian noise distribution was added to the created data set, simulating the real QEPAS signal fluctuations around the peak value. Compared with standard multilinear regression, PLSR predicted gas concentrations with a calibration error up to 5 times better, even with absorption features with spectral overlap greater than 97%.

Optical trace gas detection techniques are of great interest for a wide range of real world applications spanning from environmental protection^1,2^ and health monitoring^3,4^ to industrial process control and security,^5,6^ since they offer high sensitivity and selectivity together with fast response time. Among all optical spectroscopic techniques, quartz-enhanced photoacoustic spectroscopy (QEPAS) has emerged as a powerful, reliable, and robust technique, with demonstrated high sensitivity in the detection of several trace gas species.^7−10^ QEPAS exploits the photoacoustic effect and uses a quartz tuning fork (QTF) to detect the weak sound waves produced by molecules absorbing modulated light. Although based on a light absorption process, QEPAS works differently from direct absorption spectroscopy. The QTF signal strongly depends on the acoustic wave generation efficiency within the gas sample, which in turns is strongly related to the targeted gas molecules. For these reasons, the measured signals are not directly proportional to the line strength of the targeted absorption features. In addition, QEPAS is a wavelength-independent technique, in which the same QTF can operate with laser sources emitting in the spectral range from UV to THz. This classifies QEPAS as an ideal technique for multigas detection. Many applications require the detection of one or more analytes in a multigas mixture, specially at atmospheric pressure. Several approaches have been developed in QEPAS sensing to selectively identify different components within a mixture. One possibility is to exploit the full tunability range of a single laser source to target not-overlapped absorption features.^11^ Another approach is to employ several laser sources, each one targeting the absorption feature of a single component of the gas mixture. In this case, the light sources are shined in sequence^12^ or, for the specific instance of a two-gas mixture, simultaneously excite the QTF fundamental and first overtone resonance mode, respectively.^13−15^ QEPAS typically targets isolated absorption features to evaluate the analytes concentrations and avoid interferences from other species contained in the gas matrix. A partially resolved or unresolved spectrum, resulting from the overlap of absorption features of different gases requires a distinct approach. Multivariate analysis (MVA) is generally used to analyze and discriminate each independent analyte of a gas mixture, treated as a physical system made up of several components. The most common MVA approach is the multilinear regression (MLR), which extends the standard linear regression to multiple variables. MLR models the relationship between the concentration of each component and the measured spectra based on ordinary least-squares. An iterative fit procedure is employed to minimize the sum-of-squares of the differences between measured and predicted values, with no possibility to predict the presence of other components besides the ones used as references. This procedure is efficient as long as the experimental data, namely X-variables, are uncorrelated or at least weakly correlated, and affected by low noise. MLR shows a high statistical significance when there is no collinearity among the predictor variables. When two or more variables in a multiple regression model are correlated (multicollinearity), they cannot independently predict the value of the dependent variable, leading to a decrease in the statistical significance of the prediction. Therefore, when dealing with complex systems made of correlated data,^16^ which is the case for spectroscopic analysis of overlapping absorption features of different components in a gas mixture, these requirements cannot be guaranteed, and the use of an MLR approach can result in a lack of precision and accuracy.^17,18^ Moreover, MLR models can easily fall into overfitting problems dealing with spectroscopic data, due to the high number of involved variables.^19^ Sampaolo^20^ and Giglio^21^ detected merged absorption features using QEPAS-based sensors and analyzed using MLR. In both cases, the regression technique results in calculating values with large confidence intervals. This suggests empowering all the laser based spectroscopic techniques with a more sophisticated analysis tool whenever strongly overlapping gas species must be analyzed in a mixture. Partial Least Squares Regression (PLSR) is an excellent candidate to overcome these limitations. Originally developed as a tool for social and economic sciences,^22^ PLSR has established itself as a solid technique for modeling complex systems in physics and chemistry branches in recent years.^23−27^ PLSR extends the MLR approach to deal with a large number of strongly correlated and noisy experimental data. In this work, we combined the QEPAS technique with PLSR to identify gas components in a mixture with strongly overlapping absorption features over the full spectral dynamic range of quantum cascade laser (QCL) sources. A two-gas mixture composed of carbon monoxide (CO) and nitrous oxide (N_2_O) and a three-gas mixture of acetylene (C_2_H_2_), methane (CH_4_), and nitrous oxide have been analyzed. Both mixtures are diluted in nitrogen (N_2_). Absolute concentrations of gas components in the mixtures were estimated starting from single-gas reference spectra. Then, the results of the PLSR algorithm were compared with a standard MLR approach.

## Partial Least Squares Regression

The multiple regression equation in matrix form is as follows:
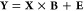
1where **X** is the *n* × *m* matrix of independent variables (matrix of experimental spectra), **Y** the *n* × *k* matrix of the predicted values of the variables (matrix of physical parameters to be estimated, i.e., the gas component concentrations), **B** is the *m* × *k* matrix of the regression coefficients, and **E** is the *n* × *k* errors matrix, assumed to be uncorrelated and with the same variance. PLSR is based on the assumption that the investigated system is influenced by a set of factors called latent variables (LVs).^28^ The prediction is achieved by extracting LVs having the best predictive power from the predictors.^18^ From a geometrical point of view, this procedure is equal to a projection of the X-variables into a new space, representative of the latent variables. The strength of the PLSR method compared to other MVA techniques (i.e., multiple linear regression, ridge regression etc.) is in the stability of predictors. Since the uncertainty of the estimated parameters is the dominant factor in the variability of predictors, it is crucial to keep the number of variables as low as possible. In this way, PLSR gives the minimum number of necessary variables.^29,30^ This technique can be used for both modeling the underlying relationship between physical or chemical parameters and performing predictive analysis on a sample with unknown properties requiring evaluation. The latter condition assumes a machine learning-like approach, where the experimental data set **X** is split into a training-set **X**_**tr**_, associated with a known **Y**_**tr**_, and a test-set **X**_**test**_, with **Y**_**test**_ to be evaluated. With the aim of evaluating the concentrations of chemical species in a multigas mixture, the training-data set will be developed starting from single-gas spectra used as reference spectra to calibrate the model and analyze the gas mixtures spectra. The PLSR analysis is performed on the training-set to calculate the regression coefficients matrix **B**, used in turn to evaluate the **Y**_**test**_ matrix via the matrix product: **Y**_**test**_ = **X**_**test**_×**B**. The regression matrix **B** provides information about the correlation between the experimental data set and the concentration of the corresponding gas, since a high absolute value of the regression coefficient highlights a significant influence of the experimental point on the gas concentration.^18^ To perform PLSR, a MATLAB code has been developed using MATLAB built-in Simple Partial Least Squares (SIMPLS) algorithm to perform the regression.^31,32^ In contrast with MLR, where the error on calibration is calculated as the error on the regression coefficients, the evaluation of the PLSR calibration error is not straightforward. A reliable tool for the estimating calibration errors is the 10-fold cross-validation (CV).^33^ This procedure returns the root mean squared error of calibration (RMSECV, ε) based on the algorithm performance in the training step, which is known a priori without any information about the test data set. Therefore, RMSECV is based on the predictive ability of the PLS algorithm rather than on the quality of the measurements under test. For these reasons, the estimation of CV-RMSEP will be considered in the following discussion as the error associated with the PLS prediction of gas concentrations in the analyzed mixtures.^34−36^ Root mean squared error of prediction (RMSEP) will be also evaluated comparing the expected concentrations and the retrieved values, for each analyte.^37^

## Experimental Section

**Figure 1:**
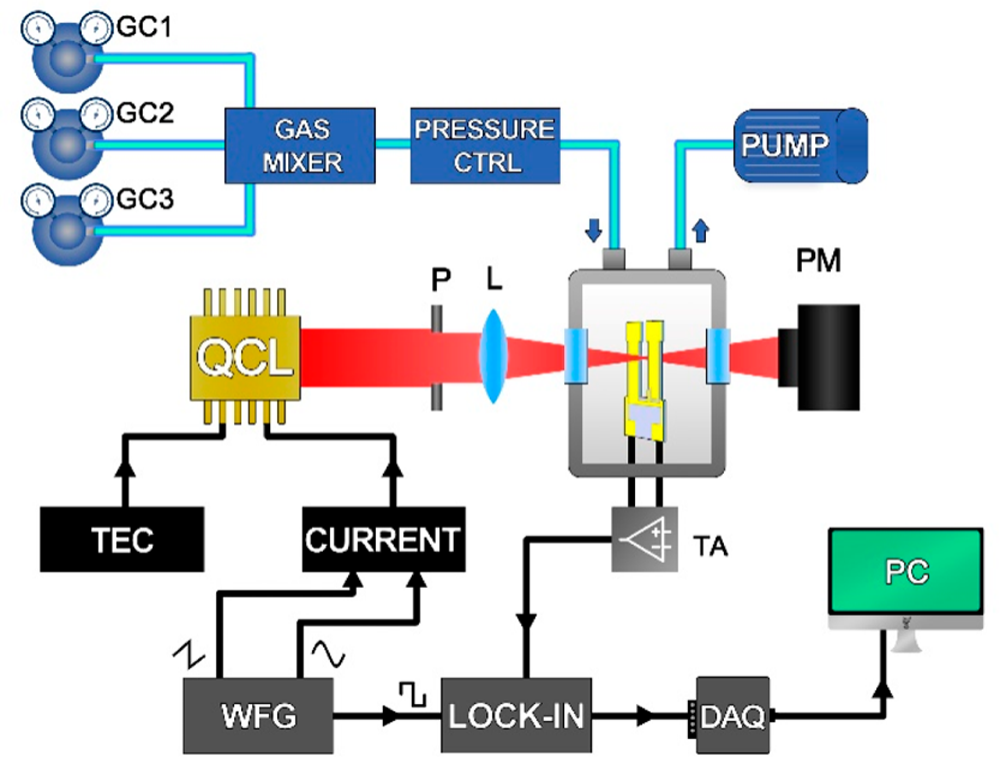
QEPAS sensor for multigas detection. QCL, quantum cascade laser; P, pinhole; L, lens; QTF, quartz tuning fork; ADM, acoustic detection module; PM, power meter; TA, transimpedance amplifier; TEC, thermo-electric cooler; WFG, waveform generator; DAQ, digital acquisition card; PC, personal computer; GC, gas cylinder; and PRESSURE CTRL, pressure controller.

The PLSR algorithm has been tested to retrieve the concentration of the single species in a two-gas mixture and a three-gas mixture. Absorption spectra of gas mixtures were acquired by using the QEPAS setup depicted in Figure 1.

An AdTech QCL with a central emission wavelength at 4.61 μm and a Corning QCL with a central emission wavelength at 7.72 μm were used to detect N_2_O and CO in a two-gas mixture and C_2_H_2_, CH_4_, and N_2_O in a three-gas mixture, respectively. For both mixtures, the absorption features can be detected by varying the laser injection current within its dynamic range, at a fixed operating temperature. The setup allowed an easy interchange of QCL sources. The laser beams were first spatially filtered by using a pinhole and then focused within an acoustic detection module (ADM) by means of a lens with focal length *f* = 75 mm.

The ADM (Thorlabs ADM01) consisted of a gas cell, equipped with two windows (Thorlabs WW01050-E1 with 2–5 μm AR coating and Thorlabs WW71050-E3 with 7–12 μm AR coating), a pair of connectors for gas inlet and outlet and a custom T-shaped quartz tuning fork with a resonance frequency of *f*_0_ = 12458 Hz and a quality factor of 12 500 at atmospheric pressure.^38^ A power meter was set behind the ADM for alignment purpose. All measurements were performed at atmospheric pressure (*P* = 760 Torr) and room temperature (*T* = 25 °C). The piezoelectric current generated by the QTF was collected and transduced into a voltage signal by a transimpedance amplifier with a feedback resistor *R*_fb_ = 10 MΩ. The voltage signal was sent to an EG&G model 7265 lock-in amplifier, set with a time constant of 100 ms. Both QCLs were polarized using an Arroyo 5300 current driver. An Arroyo 4300 thermo-electric cooler (TEC) was used to stabilize the operating temperature. QCL emission frequencies were tuned by sweeping the laser injection current with a 2 mHz triangular ramp and were simultaneously modulated by a sinusoidal waveform with frequency *f*_0_/2. The lock-in demodulated the QTF voltage signal at *f*_0_: in this way the sensor was operated in *2f based* wavelength modulation. Both the sweep and the modulation were provided by a Tektronix AFG3102 waveform generator, which also supplied the reference signal for the lock-in amplifier at *f*_0_/2. The demodulated output signal was then sent to a DAQ card (National Instrument 6002) and stored on a PC using a LabVIEW-based software. All the measurements were performed in a continuous gas flow of 30 sccm. Four cylinders with certified concentrations of the single gas targets (1000 ppm of CO in N_2_, 1000 ppm of N_2_O in N_2_, 1000 ppm of C_2_H_2_ in N_2_, and 1% of CH_4_ in N_2_) and one cylinder of pure N_2_ were used to generate different gas mixtures. A gas mixer (MCQ Instrument Gas Blender 1003) was used to manage gas flows for up to 3 different input gas lines at the same time, with 1σ single-channel accuracy of ∼1% provided by the instrument datasheet. The pressure inside the gas line was fixed and monitored by an MKS Pressure Controller Type 649.

## Results and Discussion

### Two-Gas Mixture Detection

**Figure 2:**
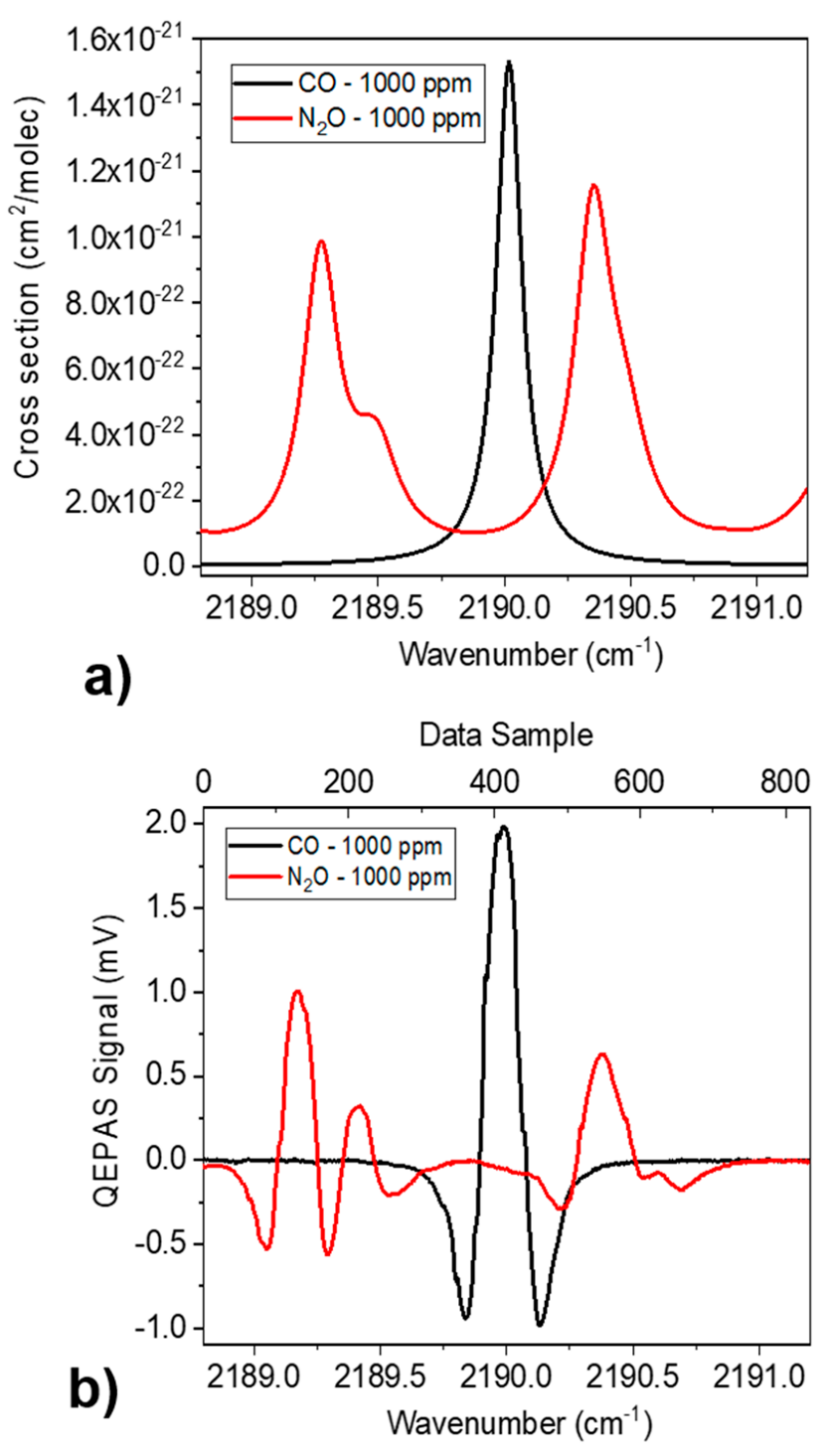
(a) HITRAN simulation of absorption cross section spectrum and (b) QEPAS scan of 1000 ppm of CO in N_2_ (black curve) and 1000 ppm of N_2_O in N_2_ (red curve).

HITRAN database^39^ was used to simulate the absorption cross section of 1000 ppm of CO in N_2_ and 1000 ppm of N_2_O in N_2_, at atmospheric pressure and room temperature over the whole spectral dynamic range of the AdTech QCL (2188.8–2191.2 cm^–1^). The results of the simulation are shown in Figure 2(a). The CO exhibits a Lorentzian-like absorption feature peaked at 2190.02 cm^–1^ while the N_2_O shows two partially merged absorption features with peaks at 2189.35 and 2189.4 cm^–1^ and a well-isolated Lorentzian-like absorption feature at 2190.35 cm^–1^.

As a first step, the single-gas reference spectra were acquired by analyzing gas mixture coming directly from the gas cylinders, without the use of the mixer. Hence, the reference spectra are referred to certified concentrations. To scan the spectral range reported in Figure 2(a), the AdTech QCL operating temperature was set at 15 °C, and the injection current was tuned from 230 mA to 310 mA. The maximum optical power measured at the injected current of 310 mA (corresponding to 2188.8 cm^–1^) was 75 mW. The QEPAS signal was collected with a lock-in amplifier demodulation phase φ_1_ = −132.17°, corresponding to the phase value maximizing the CO peak signal. The QEPAS scan referred to the positive slope of the triangular ramp is reported in Figure 2(b). In order to enlarge the data statistics, both positive and negative ramp slopes were considered in the PLSR algorithm. With a signal acquisition time of 300 ms, a single spectrum consisted in 1666 data-points. To ensure the reproducibility of the measurements, the reference data are collected every time a new set of mixtures spectra is acquired. In this way, the consistency of the operative conditions is guaranteed.

The CO reference spectrum shows a single absorption feature with a signal intensity of ∼2 mV, corresponding to the isolated absorption peak at 2190.02 cm^–1^ in Figure 2(a). From left to right, the N_2_O reference spectrum shows three features with peak intensities of ∼1, ∼0.3, and ∼0.6 mV. The first two peaks are clearly due to the partially merged absorption features at 2189.35 cm^–1^ and at 2189.4 cm^–1^, while the third peak is associated with the isolated absorption line peaked at 2190.35 cm^–1^. The 1σ-noise level measured far from the absorption features is ∼3 μV for both spectra, resulting in a Signal-to-Noise Ratio (SNR) of 660 and 330 for CO and N_2_O, respectively. The measured noise level is comparable to the calculate thermal noise value of 2.6 μV, which affects the resonator in the employed configuration.^40^

**Figure 3:**
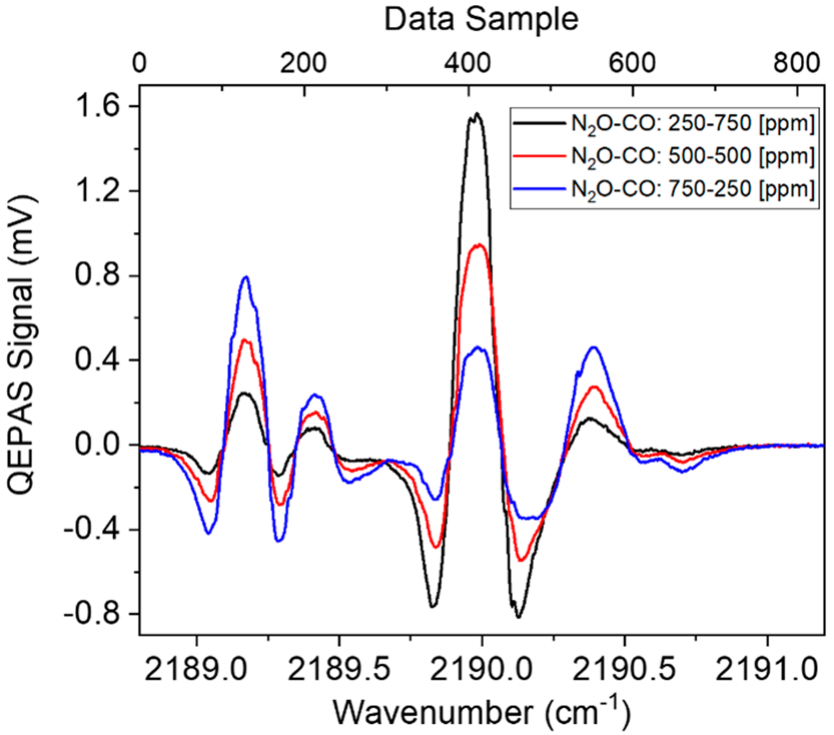
QEPAS scan acquired for three mixtures containing 250 ppm of N_2_O and 750 ppm of CO (black curve), 500 ppm of N_2_O and 500 ppm of CO (red curve), and 750 ppm of N_2_O and 250 ppm of CO (blue curve), respectively, in N_2_.

Starting from the certified concentrations of 1000 ppm of N_2_O in N_2_ and 1000 ppm of CO in N_2_, the following mixtures of N_2_O–CO were generated by using the gas blender: 250–750 ppm, 500–500, and 750–250 ppm, in N_2_. All QEPAS measurements were performed by setting the lock-in phase to φ_1_. The acquired QEPAS spectra scans are reported in Figure 3.

All absorption features of N_2_O and CO are clearly distinguishable. Spectral overlap is only limited to the superposition of the right-side negative lobe of the CO absorption feature with the left-side negative lobe of the N_2_O absorption feature peaked at 2190.35 cm^–1^.

### Two-Gas Mixture PLS Model Calibration and Test

Data analysis starts from the configuration of the training data set for PLS model calibration. MATLAB-based algorithm projects the training data set on a number of PLS factors, i.e., the number of latent variables, equal to two, representing the components of the gas mixtures. However, larger data sets correspond to lower calibration errors. Unlike MLR, PLSR allows the employment of simulated data to perform the regression.^41,42^ Hence, the experimental data set was enriched by simulated spectra calculated as linear combinations of the actual reference spectra.^25^ Actual reference spectra were combined using 10 coefficients, from 0 to 0.9 at step of 0.1, properly chosen according to the concentration range expected in mixtures under investigation. The simulation process resulted in 10^PLS-factors^ = 100 simulated spectra. This enlargement of the experimental data set represents one of the main advantages of PLSR compared to MLR. In contrast to MLR, PLSR allows for the addition of input noise fluctuations on the simulated spectra to consider the non-negligible fluctuations affecting the reference spectra. A reliable distribution of the input noise fluctuations must match the distribution of the QEPAS signal fluctuations around its mean value, namely the peak value. The experimental distribution has been retrieved by repeatedly scanning over the QEPAS absorption peaks. For both target gases, a Gaussian noise distribution with a 1σ-noise fluctuation of ∼3% around the mean value was obtained. Hence, a white Gaussian noise was superimposed to the simulated reference spectra. With these conditions, the X-training data set is a 100 × 1666 matrix (100 different simulated reference spectra, each one composed of 1666 data samples) while the Y-training data set is a 100 × 2 matrix with the related gas concentrations. A preliminary analysis on the whole data set showed that modeling the system with 2 PLS factors explains more than 99% of **Y**_**tr**_ variance, confirming the validity of the theoretical assumptions about physical relevance of PLS factors. Then, the PLSR algorithm is used to calculate regression coefficients (matrix **B**). In Table 1, the results of the PLSR applied to the three gas mixtures are reported, together with MLR results and their associated calibration errors ε. The nominal concentrations of the mixture components are also reported with an accuracy of η = ±10 ppm, calculated by considering the gas mixer flow accuracy of 1% starting from the certified gas cylinder concentrations. Considering this instrumental limitation, we used the cross-validation error ε as main indicator for quantifying the robustness of the regression model employed, i.e., PLSR and MLR.

**Table 1:**
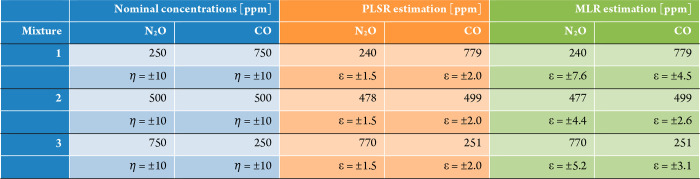
PLSR and MLR Results (Concentrations and Calibration Errors) for Each Component of Dual-Gas Mixtures^a^

a The nominal concentrations are also reported together with the accuracy determined from the gas mixer datasheet.

The results show that PLSR and MLR predict the same concentration values in gas mixtures, while the RMSECV estimated by PLSR is up to 3 times lower than the MLR estimation. The estimated values of gas concentrations are within the 2σ interval determined by the accuracy of the gas mixer. The PLSR-RMSEP are equal to 18 and 17 ppm, while the MLR-RMSEP are equal to 19 and 17 ppm, for N_2_O and CO, respectively. Due to the instrumental limitation, it is not possible to compare the collected results with the reference standard concentration values in the gas line. However, the stability of the algorithms results, which is strictly connected to the regression precision, can be verified by performing the analysis on repeated measurements. As expected from the theoretical background,^18^ the PLSR results are less affected by experimental data fluctuations. This means that bias effects in concentrations estimation can be removed in a validation step to be performed before moving the sensor outside the laboratory.

**Figure 4:**
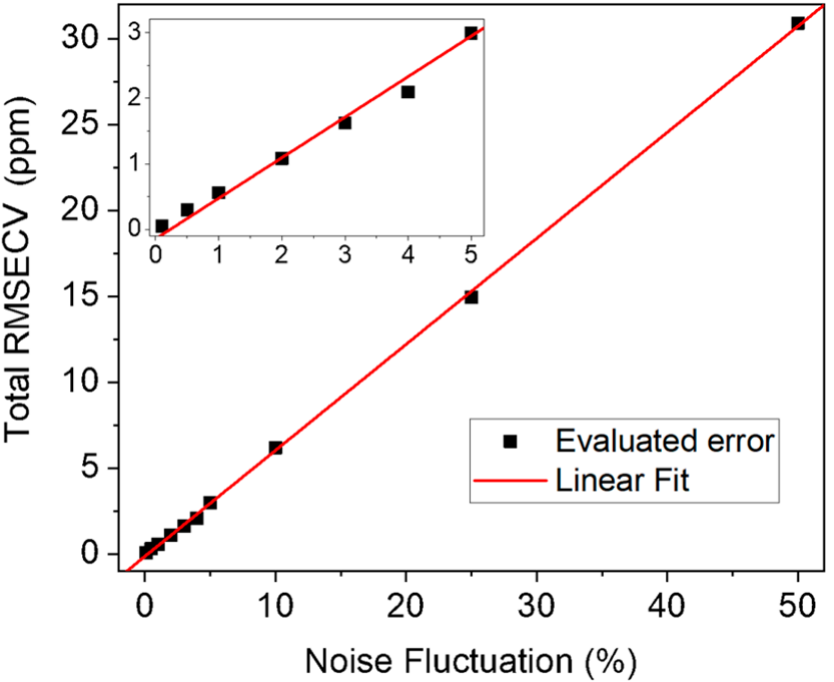
Total RMSECV as a function of the 1σ-noise fluctuation added to simulated spectra in training data set (black squares) and the best linear fit (red line). Inset: zoom in the range 0–5% of noise fluctuations, as typical values in spectroscopic experiments.

The influence of the input noise fluctuations added to the simulated spectra on the calibration error has been evaluated. PLSR analysis was performed by varying the input 1σ-noise fluctuation to evaluate the effect both on the retrieved concentrations and on the associated errors ε. Negligible variations in the estimated concentration values (<1 ppm) were calculated for fluctuations up to 50%. Whereas, the ε values are strongly dependent from input noise fluctuations. Figure 4 shows the total RMSECV, calculated as the square root of the sum of the squared ε of the single gases divided by the number of gases, as a function of input 1σ-noise fluctuations.

Equation *y* = (0.617 ± 0.004)*x* + (−0.14 ± 0.07) with *R*^2^ = 0.999 is the best fit for the data in Figure 4.

### Three-Gas Mixture Detection

**Figure 5:**
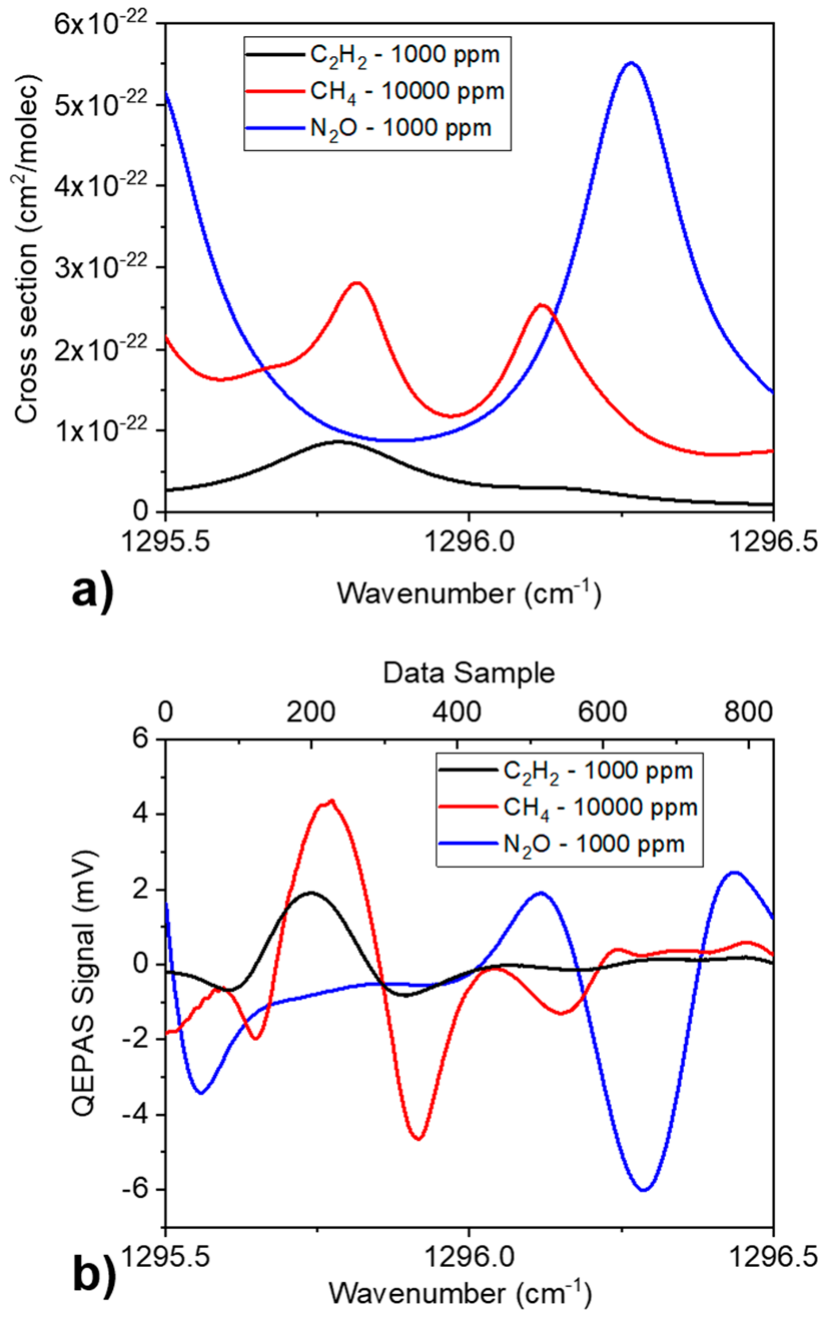
(a) HITRAN simulation of the absorption cross section spectrum and (b) QEPAS scan of 1000 ppm of C_2_H_2_ in N_2_ (black curve), 10 000 ppm of CH_4_ in N_2_ (red curve), and 1000 ppm of N_2_O in N_2_ (blue curve).

Gas mixtures with three components having a strong spectral overlap were tested to get a benchmark on the efficiency of PLSR in analyzing QEPAS-based absorption features. With this aim, C_2_H_2_, CH_4_, and N_2_O were selected. Figure 5(a) shows HITRAN database simulations^39^ at atmospheric pressure and room temperature of the listed gases absorption cross-section within the emission spectral range of the Corning QCL operated at 30 °C, when varying the injection current from 200 to 270 mA (1295.5 cm^–1^- 1296.5 cm^–1^). The cross-sections are scaled on the certified concentrations in gas cylinders: 1000 ppm for C_2_H_2_, 1000 ppm for N_2_O, and 10 000 ppm for CH_4_, in N_2_. C_2_H_2_ has a strong absorption features peaked at 1295.78 cm^–1^ and a weak one at 1296.16 cm^–1^; CH_4_ has two absorption lines falling at 1295.81 and 1296.12 cm^–1^; and N_2_O shows a single absorption line peaked at 1296.27 cm^–1^. The maximum optical power detected at the injected current of 270 mA is 112 mW. To build the training data set, the single-gas reference spectra for the three target gases were acquired directly from the gas cylinders. The lock-in amplifier demodulation phase was fixed at φ_2_ = −136.75°, corresponding to the phase maximizing the C_2_H_2_ peak signal. This choice allowed the enhancement of the C_2_H_2_ spectral feature, showing the weakest absorption coefficient. The three QEPAS spectral scans obtained by sweeping the QCL injection current are reported in Figure 5(b). As for the two-gas mixtures, the reference data are collected every time a new set of mixtures spectra is acquired, in order to ensure the consistency of the operative conditions.

The C_2_H_2_ reference spectrum shows a characteristic line-shape of the second derivative of Lorentzian profile, with a signal intensity of ∼1.9 mV. Due to the choice of lock-in demodulation phase, the spectral characteristics of N_2_O have the same line-shape of C_2_H_2_ but inverted. The QEPAS CH_4_ reference spectrum has a pronounced absorption peak of ∼4.3 mV, corresponding to the strongest absorption peak at 1295.81 cm^–1^, while the absorption feature at 1296.12 cm^–1^ is also recognizable but inverted in shape due to a difference in signal phase with the peak at 1295.81 cm^–1^. On the left side of the graph, CH_4_ and C_2_H_2_ strongly overlap; on the right side, the N_2_O absorption feature is weakly disturbed by the other two gases. The measured 1σ-noise is ∼4 μV for all three gases, comparable with the QTF thermal noise and resulting in an SNR of 470, 1150, and 1500 for C_2_H_2_, CH_4_, and N_2_O, respectively.

**Figure 6:**
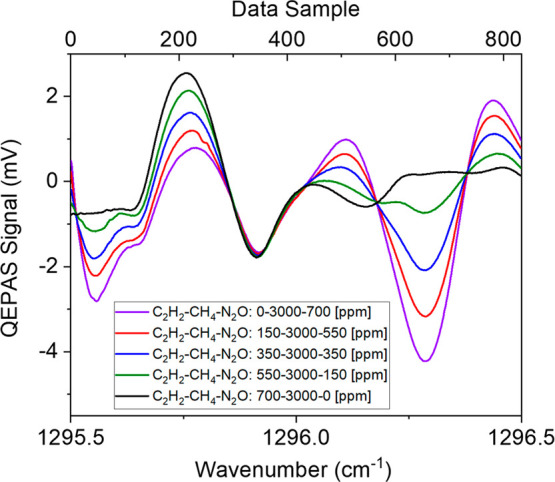
QEPAS scan for five mixtures containing 0 ppm of C_2_H_2_, 3000 ppm of CH_4_ and 700 ppm of N_2_O (purple curve), 150 ppm of C_2_H_2_, 3000 ppm of CH_4_ and 550 ppm of N_2_O (green curve), 350 ppm of C_2_H_2_, 3000 ppm of CH_4_ and 350 ppm of N_2_O (blue curve), 550 ppm of C_2_H_2_, 3000 ppm of CH_4_ and 150 ppm of N_2_O (red curve), 700 ppm of C_2_H_2_, 3000 ppm of CH_4_, and 0 ppm of N_2_O (black curve).

Starting from the certified concentrations, five mixtures of C_2_H_2_–CH_4_–N_2_O, with a fixed concentration of 3000 ppm of CH_4_ have been generated, as reported in the legend of Figure 6. All the QEPAS measurements were performed by setting the lock-in phase to φ_2_. The acquired QEPAS spectra scans are reported in Figure 6.

As expected, the strong absorption feature of N_2_O is well recognizable at 1296.27 cm^–1^(650 data sample). The CH_4_ and C_2_H_2_ absorption features are completely overlapped in the wavenumber range from 1295.65 to 1295.91 cm^–1^, while deformations of the spectra induced by the increasing amount of acetylene can be observed in the 1295.50 cm^–1^– 1295.65 cm^–1^ sample range.

### Three-Gas Mixture PLS Analysis

The PLSR has been performed by projecting the training data set on three PLS factors, representing the number of gas components in the mixtures. As for the two-gas mixture, a 1000 × 1666 matrix **X**_**tr**_, has been obtained by simulating 10^PLS-factors^ = 1000 spectra with a superimposed Gaussian noise. The Y training data set is a 1000 × 3 matrix with the associated gas concentrations. Preliminary analysis on the whole data set shows that modeling the system with 3 PLS factors explains more than 99% of **Y**_**tr**_ variance. The PLSR is therefore performed, and the regression coefficients matrix **B** is calculated. As for the two-gas mixture analysis, variations lower than 1 ppm in the estimated concentration values were calculated for input 1σ-noise fluctuations up to 50%. The analysis of the Total RMSECV as a function of Gaussian noise fluctuation showed a linear trend with a best fit equation *y* = (4.65 ± 0.04)*x* + (−0.61 ± 0.64) and *R*^2^ = 0.999. In Table 2, the PLSR results for the five mixtures shown in Figure 6 and related MLR results are reported.

**Table 2:**
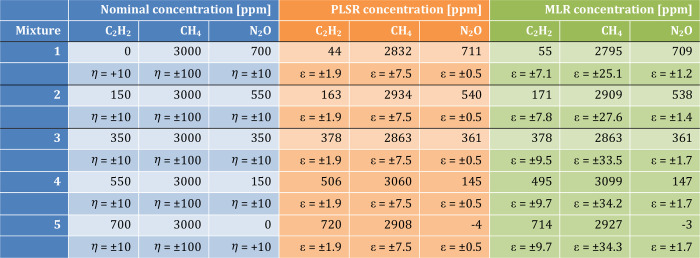
PLSR and MLR Results (Concentrations and Calibration Errors) for Each Component of the Analyzed Three-Gas Mixtures^a^

a The nominal concentrations are also reported together with the accuracy determined by the gas mixer datasheet.

In contrast to the two-gas mixtures analysis, PLSR and MLR predict different concentration values. The estimated values of gas concentrations are within the 2σ interval determined by the accuracy of the gas mixer, with few exceptions for C_2_H_2_. This can be ascribed to the difficulties of both methods in the identification of the C_2_H_2_ contribution, due to the strong overlap with the CH_4_ absorption line, as supported by the highest relative error measured for C_2_H_2_ in all mixtures. With three-gas mixtures with strongly overlapped features, calibration error by PLSR is significantly lower than MLR, up to a factor of ∼5. The calculated PLSR-RMSEP are equal to 32, 113, and 9 ppm, while the MLR-RMSEP are equal to 39, 130, and 9 ppm for C_2_H_2_, CH_4_, and N_2_O, respectively. In mixture 1 with no C_2_H_2_, both PLSR and MLR predict the presence of C_2_H_2_, with a concentration of 44 and 55 ppm, respectively. A lower accuracy for the MLR can be explained considering that the algorithm is forced to search for all the gas components set as reference spectra. Therefore, a higher bias in regression is expected, reducing the accuracy of the prediction. However, as reported for the two-gas mixtures, even with three-gas mixtures, the PLSR results were verified to be more stable to repeated measurements compared to MLR ones. The evidence of the bias influence can be observed repeating the analysis excluding the C_2_H_2_ reference spectrum from the training data set. The retrieved concentrations thus become 2958 and 710 ppm for CH_4_ and N_2_O, respectively, and a decrease in calibration error is obtained, with ε_CH4_ = 4.2 ppm and ε_N2O_ = 0.4 ppm. In the mixture with no N_2_O, both methods predict a negative value for N_2_O concentration: this is obviously not possible and must be intended as a zero concentration. However, with respect to C_2_H_2_ estimation in the mixture with no C_2_H_2_, both algorithms are more accurate in the prediction because the N_2_O absorption feature is well-defined within all mixture spectra.

These kinds of false-positive results may occur when dealing with missing components, as well as false-negative results may occur when one of the target analytes generates a negligible QEPAS signal. For real-field applications, regression algorithms are trained on analyte concentrations similar to the ones expected in the sample to test. In this case, a threshold concentration can be set to discern the effective presence of a chemical species, based on the expected sample composition.

### Overlap Parameter Estimation

The results obtained for the two-gas mixtures showed that, when dealing with weakly overlapping spectral features, the PLSR and the MLR return the same values, but the PLSR calibration error estimation can be up to 3 times lower than that of the MLR. When analyzing spectra originated by strongly overlapping absorbing features, PLSR predicts different gas concentrations with respect to MLR, with a lower calibration error up to a factor of 5. To quantify the overlap between absorption features, a parameter should be introduced. Considering the Lorentzian-like line-shape (see Figures 2(a) and 5(a)), the overlap parameter *Z* between two absorption features labeled as 1 and 2 can be defined as follows:
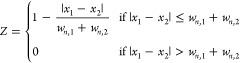
2where *x*_*i*_ is the peak wavenumber, and *w*_*n,i*_ is the normalized Lorentzian width defined as the ratio between the full-width-half-maximum of the Lorentzian curve *w*_*i*_ and the peak value *A*_*i*_.

The overlap parameter tends to 0 when the distance between the absorption peaks tends to *w*_*n*__,1_ + *w*_*n*__,2_, while *Z* is equal to 1 when *x*_1_ = *x*_2_. For features whose peaks distance is greater than *w*_*n*__,1_ + *w*_*n*__,2_, *Z* is negative and overlap effects are negligible. The overlap parameters (in %) calculated for adjacent absorption peaks in the three-gas mixture are *Z*_CO-N2O_ = 7.3%, *Z*_CH4-N2O_ = 79.8%, and *Z*_C2H2-CH4_ = 97.4%. With overlap as high as 97%, PLSR is able to identify both contributions with a precision significantly higher than that of the standard MLR.

## Conclusions

In this work, we combined QEPAS with PLSR analysis to retrieve single components gas concentrations in multigas samples. Two different mixtures have been analyzed, one composed of two gases (CO–N_2_O) and the other one composed of three gases (C_2_H_2_–CH_4_–N_2_O), both diluted in N_2_. A QEPAS sensor has been realized using a custom quartz tuning fork and employing two QCLs emitting at 4.61 and 7.72 μm for the two- and three-gas mixture investigation, respectively. As a first step, the single-gas reference spectra were acquired. The PLSR procedure was implemented using a training-test approach. The training data set was built starting from the reference spectra and was enlarged by means of simulated spectra, calculated as linear combinations of reference ones, exploiting the ability of PLS model to deal with correlated measurements. A Gaussian distribution noise was added to the simulated spectra to consider the experimental errors involved in the measurements. PLSR calibration errors have been calculated as a cross-validation error on the training data set. Then, the PLSR algorithm was employed to retrieve gas concentrations in a series of gas mixtures generated from certified single-gas concentrations. The estimated values of gas concentrations are within the 2σ interval determined by the accuracy of the gas mixer. Compared to MLR, the error of calibration decreases by a factor of ∼3, for CO–N_2_O mixtures, and up to a factor of ∼5, for C_2_H_2_–CH_4_–N_2_O mixtures. To properly quantify the superposition among the spectral features, an overlap parameter was defined by considering the distance between the spectral peaks and their width. This allowed us to affirm that PLSR can identify a single-gas contribution in a mixture even when a 97% spectral overlap occurs.

Further applications of the PLSR approach can involve the analysis of gas mixtures with missing components (like mixture 1 in Table 2) exploiting the PLS capability of finding the number of components in the training step. The mutual influence of the analytes, as in the case of species acting as promoters, could also be estimated. The next step will be the testing of the sensor outdoor, in this case the concentration of water vapor acting as relaxation promoter must be fixed by using a Nafion humidifier.^13^ The ADM will also be heated up to 40 °C to avoid adsorption of sticky molecules like H_2_O and NH_3_ on the internal surfaces of the sensor.^43^

## Abbreviations Used

QEPAS: quartz-enhanced photoacoustic spectroscopy
QTF: quartz tuning fork
QCL: quantum cascade laser
MLR: multilinear regression
PLS: partial least-squares
PLSR: partial least-squares regression
